# Short-term effectiveness of dapagliflozin versus DPP-4 inhibitors in elderly patients with type 2 diabetes: a multicentre retrospective study

**DOI:** 10.1007/s40618-022-02002-2

**Published:** 2023-01-09

**Authors:** M. L. Morieri, I. Raz, A. Consoli, M. Rigato, A. Lapolla, F. Broglio, E. Bonora, A. Avogaro, G. P. Fadini, for the DARWIN-FUP network, Federica Ginestra, Gloria Formoso, Agostino Consoli, Francesco Andreozzi, Giorgio Sesti, Salvatore Turco, Luigi Lucibelli, Adriano Gatti, Raffaella Aldigeri, Alessandra Dei Cas, Giuseppe Felace, Patrizia Li Volsi, GianPio Sorice, Andrea Giaccari, Carmen Mignogna, Raffaella Buzzetti, Tiziana Filardi, Susanna Morano, Ilaria Barchetta, Maria Gisella Cavallo, Ilaria Malandrucco, Simona Frontoni, Silvia Carletti, Paola D’Angelo, Gaetano Leto, Frida Leonetti, Paola Silvia Morpurgo, Paolo Fiorina, Eva Palmieri, Emanuela Orsi, Enzo Mantovani, Ivano Franzetti, Fabrizio Querci, Antonio Bossi, Federica Turchi, Silvana Manfrini, Danila Guida, Giuseppe Placentino, Guglielmo Beccuti, Fabio Broglio, Franco Cavalot, Alessandro Nuzzo, Gianluca Aimaretti, Olga Lamacchia, Angelo Cignarelli, Luigi Laviola, Francesco Giorgino, Eleonora Devangelio, Giuliana Cazzetta, Roberta Chianetta, Roberto Citarrella, Andrea Tumminia, Lucia Frittitta, Massimiliano Anzaldi, Massimo Buscema, Salvatore Piro, Antonino Di Pino, Francesco Purrello, Antonino Di Benedetto, Giuseppina Russo, Roberto Anichini, Anna Solini, Monia Garofolo, Stefano Del Prato, Bruno Fattor, Gian Paolo Fadini, Angelo Avogaro, Annunziata Lapolla, Giovanni Sartore, Michele D’Ambrosio, Virgilio Da Tos, Vera Frison, Natalino Simioni, Massimo Cigolini, Enzo Bonora, Elisabetta Brun, Marco Strazzabosco, Maurizio Poli, Mauro Rigato, Agostino Paccagnella, Carmela Vinci

**Affiliations:** 1 Department of Medicine, University of Padova, Via Giustiniani 2, 35128 Padua, Italy; 2 Department of Medicine, Hadassah Hebrew University Hospital, Jerusalem, Israel; 3 DMSI & CAST, University G. d’Annunzio of Chieti-Pescara, Chieti, Italy; 4 Diabetology Clinic, AULSS2 Marca Trevigiana, 31100 Treviso, Italy; 5 Department of Medical Sciences, University of Turin, 10124 Turin, Italy; 6 Department of Medicine, University and Hospital Trust of Verona, Verona, Italy

**Keywords:** Observational, Cardiovascular, Heart failure, Kidney disease, Aging

## Abstract

**Aim:**

To compare effectiveness of dapagliflozin versus DPP-4 inhibitors on individualized HbA1c targets and extra-glycaemic endpoints among elderly patients with type 2 diabetes (T2D).

**Methods:**

This was a multicentre retrospective study on patients aged 70–80 years with HbA1c above individualized target and starting dapagliflozin or DPP-4 inhibitors in 2015–2017. The primary outcome was the proportion reaching individualized HbA1c targets. Confounding by indication was addressed by inverse probability of treatment weighting (IPTW), multivariable adjustment (MVA), or propensity score matching (PSM).

**Results:**

Patients initiating dapagliflozin (*n* = 445) differed from those initiating DPP-4i (*n* = 977) and balance between groups was achieved with IPTW or PSM. The median follow-up was 7.5 months and baseline HbA1c was 8.3%. A smaller proportion of patients initiating dapagliflozin attained individualized HbA1c target as compared to those initiating DPP-4 inhibitors (RR 0.73, *p* < 0.0001). IPTW, MVA, and PSM yielded similar results. Between-group difference in the primary outcome was observed among patients with lower eGFR or longer disease duration. Dapagliflozin allowed greater reductions in body weight and blood pressure than DPP-4 inhibitors.

**Conclusions:**

Elderly patients with T2D initiating dapagliflozin had a lower probability of achieving individualized HbA1c targets than those initiating DPP-4 inhibitors but displayed better improvements in extra-glycaemic endpoints.

**Supplementary Information:**

The online version contains supplementary material available at 10.1007/s40618-022-02002-2.

## Introduction

The population of western countries is progressively aging. As the prevalence of diabetes increases with age, a large proportion of people with diabetes falls into elderly categories, often > 70 years old [1]. Treatment of diabetes in such elderly group has specific needs related to targets, complications, and adverse events [2]. The aging diabetic population exhibits particularly high rates of heart failure (HF) and kidney disease, both acute and chronic [3]. Prevention of these conditions is key to improve quality of life in aged people with diabetes. Trials with sodium glucose cotransporter-2 inhibitors (SGLT2i) have demonstrated extensive benefits on multiple hard endpoints in diversified populations [4]. Among individuals with type 2 diabetes (T2D), SGLT2i reduced the rates of hospitalization for HF, the progression of chronic kidney disease (CKD), and the development of acute kidney injury (5; 6). Among people with a history of HF or CKD and with or without T2D, SGLT2i improved overall outcomes [7–9]. Based on these effects, SGLT2i appear particularly suited for the treatment of elderly people with diabetes. However, diabetic patients aged 70 years or older represented a minority of those enrolled in phase III trials and studies examining whether SGLT2i maintain their effects in aging individuals with acceptable safety and tolerability profiles are scant. According to a re-analysis of the DECLARE trial, dapagliflozin maintained its glucose-lowering efficacy and was equally effective in preventing cardiovascular death or hospitalization for heart failure in patients aged < 65 years, 65–75 years, or > 75 years [10]. Nonetheless, clinicians may be concerned with some rare side effects of SGLT2i, like volume depletion, that may be particularly dangerous in the elderly. In Italy, there has been caution in adopting SGLT2i treatment in the individuals with T2D age > 70 years, mainly in favour of dipeptidyl-peptidase-4 inhibitors (DPP-4i) [3]. In the interval trial, people with T2D aged 70 years or older who were randomized to vildagliptin versus placebo exhibited a threefold higher probability of reaching their individualized HbA1c target, without new safety signal [11]. Yet, it is questionable that DPP-4i are preferable over SGLT2i in elderly patients, since DPP-4i exert no protection against cardio-renal complications [12, 13].

In older patients, glycaemic targets need to be adjusted to the degree of frailty and life expectancy, such that the use of individualized targets is recommended. In 2015, based on a consensus among key worldwide opinion-leading diabetologists, Cahn et al. proposed an algorithm for calculating the individualized glycaemic goals in patients with T2D [14]. There is a general paucity of studies specifically focusing on the elderly population, even in the real-world evidence (RWE) setting, and the adoption of individualized glycaemic targets is still uncommon.

In this study, we aimed to compare the probability of attaining individualized HbA1c target among elderly patients with T2D who initiated the SGLT2i dapagliflozin or a DPP-4i under specialist care in Italy.

## Methods

### Study design

DARWIN-FUP (dapagliflozin real-world evidence follow-up) was a retrospective multicentre study conducted at 56 diabetes specialist outpatient clinics in Italy. The study collected data on patients who received for the first time a prescription of dapagliflozin or a DPP-4 inhibitor from 2015 to 2017. In the study period, SGLT2i and DPP4i could be prescribed only by diabetes specialists. The primary objective was comparing the effectiveness of dapagliflozin versus DPP-4 inhibitors on a composite endpoint of HbA1c, body weight, and blood pressure reduction. Details on the study design, along with results of such primary analysis, have been published elsewhere [15]. The protocol was approved by the Ethical Committees of all participating centres. In agreement with the National regulations on observational studies, the need for informed consent was waived.

### Cohort identification

In this secondary analysis of the DARWIN-FUP study, we included only data on male and female patients with a diagnosis of type 2 diabetes since at least 1 year, aged 70–80 years, who received first prescription of dapagliflozin or DPP4i on top of metformin with or without insulin and had at least one follow-up visit available for the evaluation of effectiveness. The lower limit of 70 years was chosen to match a more modern definition of “elderly” as opposed to the traditional threshold of 65 years, accounting for population aging, as suggested by the United Nations [16]. The upper limit of 80 years was defined by the DARWIN-FUP protocol [15]. General exclusion criteria applied in the entire dataset, such as other forms of diabetes, chronic kidney disease stage III or higher (which was a contraindication to prescription of SGLT2i), and prior use of dapagliflozin or DPP4i. We selected only patients with HbA1c levels above the personalized target, defined accordingly to the short form previously described [14], which is based on life expectancy, disease duration, hypoglycaemia risk from treatment, comorbidities, and complications. Life expectancy was based on age- and sex-adjusted national survival curves [17], whereas the degree of comorbidity was inferred from the number of concomitant medications other than diabetes drugs.

### Data collection

Data were extracted automatically from the same electronic chart system at all centres. The baseline date was set as the date patients received the first prescription of dapagliflozin, or DPP4i, whereas follow-up was collected at the last routine visit at the same clinic, at least 3 months but less than 12 months after baseline. At baseline, we collected information on demographics, anthropometrics, risk factors, laboratory values, complications, and therapies (for details, see [15]). At the follow-up date, we collected endpoint data to evaluate effectiveness, i.e., HbA1c, body weights, systolic blood pressure, and whether the patients continued receiving dapagliflozin or stopped the drug.

### Endpoints

The primary endpoint of this analysis was the proportion of patients achieving the individualized HbA1c target. Secondary outcomes were the change in HbA1c, body weight, and systolic blood pressure.

### Statistical analysis

Continuous data are presented as mean and standard deviation (SD), whereas categorical variables are shown as percentages. Non-normal variables upon the Kolmogorov–Smirnov test were log-transformed before analysis with parametric tests. The comparison of baseline characteristics between the two groups was performed using Student’s t test for continuous variables and the Chi-square test for categorical variables. The within-group changes in endpoint variables was assessed using the paired Student’s t test with two tails.

We used three different approaches to control the confounding by indication (channelling bias), as depicted in Figure S1. In the primary analysis, we used the inverse probability of treatment weighting (IPTW), estimating a propensity score (PS) for the probability of being treated with dapagliflozin. Propensity scores (PS) were calculated from the following baseline covariates: age, sex, duration of diabetes, baseline body weight, systolic and diastolic blood pressure, HbA1c, HbA1c target, fasting plasma glucose, total and HDL cholesterol, triglycerides, eGFR, micro- or macro-albuminuria, diabetic retinopathy, diabetic macular oedema, microangiopathy, macroangiopathy, carotid atherosclerosis, history of stroke/TIA, coronary revascularization, ischemic heart disease (IHD), coronary heart disease (CHD), history of heart failure, left-ventricular hypertrophy (LVH), use of other GLM (metformin and insulin), and other medications (angiotensin-converting enzyme inhibitors or angiotensin receptor blockers, calcium channel blockers, anti-platelet therapies, beta-blockers, diuretics, and statins). To reduce bias arising from immortal time and time lag, we also included in PS models the number of GLM classes used by the patients before starting DPP-4i or dapagliflozin and the calendar year of index date. The residual of imbalances of the IPTW analyses was evaluated comparing the weighted SMD between dapagliflozin and DPP4i group (i.e., SMD ≥ 0.1 and *p* ≤ 0.05). Direct comparison of the outcome was allowed when there was no residual imbalance. Thus, the proportion of patients meeting the primary and secondary endpoints were compared with log-binomial regression or linear regression model without any further adjustment, or with further adjustment in case of residual imbalances.

We performed sensitivity analyses in the entire dataset by means of multivariable adjusted (MVA) linear or log-binomial regression models (or, whenever the latter failed to converge, using Poisson regression model with robust error variances). These MVA analyses were adjusted for all clinical characteristics used to compute PS, as listed above. Additional sensitivity analysis was performed on the primary outcome after 1:1 propensity score matching (PSM) based on the same PS used in the IPTW analysis.

For IPTW, PSM, and MVA, full datasets of baseline variables were needed to compute PS or to be entered in the regression models. Therefore, missing data were handled with multiple imputation (MI). MI was performed as previously described [18], with a fully conditional specification (FCS) algorithm [19] and obtaining ten imputed datasets including only covariates with less than 50% of missing values. Outcome variables were not imputed. Outcome analyses with IPTW and MVA were performed on each imputed dataset and pooled estimated treatment difference (ETD) are presented [20]. Relative risk (RR) with 95% confidence interval (CI) was calculated for binomial outcomes.

The primary analyses were conducted following an intention to treat (ITT) approach (i.e., including all patients regardless of whether they continued to be prescribed such treatment at follow-up). Additional sensitivity analyses were conducted in the “as-treated” (AT) dataset, including only patients for whom the prescription of DPP-4i or dapagliflozin was confirmed at the follow-up visit. Information on reasons for stopping or on drug refills rates was not available to evaluate adherence.

All analyses were stratified by sex, disease duration (< > 15 years), body mass index (< > 30 kg/m^2^), baseline HbA1c (< > 8.5%), eGFR (quartiles), concomitant insulin treatment, history of major adverse cardiovascular events (MACE), and formal interaction analyses were performed to evaluate whether ETD was influenced by these possible moderators.

A two-tailed *p* value < 0.05 was considered statistically significant. Statistical analyses were performed using SAS version 9.4 (TS1M4), and graphs were produced with GraphPad Prism ver. 8.

## Results

### Patients’ characteristics

From an initial population of 396,846 patients with type 2 diabetes followed at 56 specialist outpatient clinics, we identified 6,334 who initiated dapagliflozin or DPP4i between 2015 and 2017, 4015 of whom had follow-up information for one or more of the elected endpoints. Among them, 1422 patients were aged 70–80 years and had HbA1c levels above individualized targets and were finally included in this analysis (Fig. S1). Patients (53.4% men) were on average 74.2 years old and had a median diabetes duration of 13 years. Mean BMI was 29.3 kg/m^2^ and baseline HbA1c was 8.3% (67 mmol/mol). 92.8% of patients were receiving metformin and 37.6% were on insulin. Micro- and macroangiopathy were present in 32.7% and 37.5% of patients, respectively, with 18.6% having a history of MACE. As shown in Table 1, 455 and 977 patients were treated with dapagliflozin and DDP4i, respectively. Patients initiating dapagliflozin were younger, with longer duration of diabetes, worse glycaemic, and blood pressure control and with higher prevalence of microvascular complications and use of insulin.

**Table 1:** Clinical characteristics of study patients

	Avail (%)	Before IPTW				After IPTW			
		DPP4i *N* = 977	Dapagliflozin *N* = 445	SMD	*p*	DPP4i *N* = 977	Dapagliflozin *N* = 445	SMD	*p*
Year visit	100%	2016 (2015—2017)	2017 (2016—2017)	0.24	< .0001	2016 (2016—2017)	2016 (2016—2017)	0.03	0.50
Age, years	100%	74.7 ± 3.1	73.1 ± 2.7	0.54	< .0001	74.2 ± 3.7	74.1 ± 5.5	0.03	0.41
Sex male, *n* (%)	100%	523 (53.5%)	236 (53.0%)	0.01	0.86	532 (54.5%)	252 (56.5%)	0.04	0.36
Diabetes duration, years	100%	12 (7—18)	16 (10—21)	0.30	< .0001	13 (8—18)	12 (7—19)	0.01	0.75
Weight, kg	89%	75.5 ± 14.2	86.1 ± 15.5	0.72	< .0001	77.9 ± 18.3	79.7 ± 25.0	0.08	0.03
BMI, kg/m^2^	87%	28.1 ± 4.6	31.7 ± 5.4	0.73	< .0001	28.9 ± 5.9	29.4 ± 8.5	0.08	0.05
SBP, mm Hg	72%	139.5 ± 19.0	144.6 ± 20.3	0.26	< .0001	140.7 ± 22.9	139.7 ± 32.5	0.03	0.38
DBP, mm Hg	72%	76.8 ± 8.8	77.7 ± 10.3	0.10	< .0001	76.9 ± 10.7	76.8 ± 16.7	0.01	0.80
FPG, mg/dl	89%	156.3 ± 38.6	180.0 ± 58.6	0.48	< .0001	164.4 ± 57.7	163.5 ± 83.3	0.01	0.74
HbA1c, %	100%	8.0 ± 0.8	8.9 ± 1.2	0.83	< .0001	8.3 ± 1.4	8.3 ± 1.9	0.01	0.81
Target HbA1c, (%)	100%	7.1 ± 0.4	7.3 ± 0.4	0.74	< .0001	7.1 ± 0.4	7.1 ± 0.7	0.02	0.67
At target, *n* (%)	100%	0 (0.0%)	0 (0.0%)			0 (0.0%)	0 (0.0%)		
Total cholesterol, mg/dl	73%	166.3 ± 35.6	169.7 ± 34.4	0.10	0.15	167.1 ± 44.4	165.3 ± 60.5	0.03	0.40
HDL cholesterol, mg/dl	70%	51.1 ± 13.5	48.7 ± 13.4	0.18	0.01	50.4 ± 16.3	50.4 ± 25.4	0.00	0.97
Triglycerides, mg/dl	72%	113 (83—153)	127 (94—185)	0.33	< .0001	117 (85—161)	119 (83—159)	0.01	0.84
LDL cholesterol, mg/dl	69%	90.0 ± 30.2	91.2 ± 28.7	0.04	0.57	90.8 ± 36.8	87.4 ± 48.4	0.08	0.12
eGFR, ml/min/1.73 m^2^	100%	79.1 ± 10.3	80.2 ± 10.2	0.11	0.06	79.8 ± 12.4	80.1 ± 17.5	0.02	0.58
Complications									
CKD III stage, *n* (%)	100%	0 (0.0%)	0 (0.0%)			0 (0.0%)	0 (0.0%)		
Nephropathy, *n* (%)	100%	114 (11.7%)	84 (18.9%)	0.20	0.0003	131 (13.4%)	60 (13.5%)	0.00	0.94
Retinopathy, *n* (%)	81%	83 (10.5%)	85 (23.2%)	0.34	< .0001	100 (10.2%)	53 (11.9%)	0.05	0.19
DME, *n* (%)	81%	8 (1.0%)	20 (5.4%)	0.25	< .0001	15 (1.5%)	9 (2.0%)	0.04	0.36
Carotid ather., *n* (%)	58%	213 (37.6%)	94 (37.3%)	0.01	0.93	260 (26.6%)	115 (25.9%)	0.02	0.71
Stroke/TIA, *n* (%)	58%	51 (9.0%)	23 (9.1%)	0.00	0.96	56 (5.7%)	21 (4.8%)	0.04	0.28
Prior MI, *n* (%)	86%	42 (5.1%)	20 (5.1%)	0.00	1.00	48 (4.9%)	19 (4.3%)	0.03	0.54
Coronary Revasc., *n* (%)	86%	64 (7.8%)	33 (8.4%)	0.02	0.70	68 (7.0%)	25 (5.6%)	0.06	0.17
CHD, *n* (%)	86%	95 (11.5%)	46 (11.7%)	0.01	0.92	105 (10.7%)	39 (8.8%)	0.06	0.15
Heart failure, *n* (%)	86%	15 (1.8%)	13 (3.3%)	0.09	0.10	22 (2.2%)	13 (3.0%)	0.05	0.33
LVH, *n* (%)	86%	76 (9.2%)	52 (13.2%)	0.13	0.03	93 (9.6%)	41 (9.2%)	0.01	0.79
Microangiopathy, *n* (%)	100%	276 (28.2%)	189 (42.5%)	0.30	< .0001	300 (30.7%)	142 (31.9%)	0.02	0.53
Macroangiopathy, *n* (%)	89%	324 (37.5%)	150 (37.5%)	0.00	0.99	352 (36.0%)	149 (33.5%)	0.05	0.23
Established CVD, *n* (%)	89%	161 (18.7%)	74 (18.5%)	0.00	0.95	168 (17.2%)	64 (14.4%)	0.08	0.07
Diabetes medications^a^									
Insulin, *n* (%)	100%	250 (25.6%)	285 (64.0%)	0.84	< .0001	353 (36.2%)	165 (37.1%)	0.02	0.64
Metformin, *n* (%)	100%	938 (96.0%)	382 (85.8%)	0.36	< .0001	922 (94.4%)	414 (93.0%)	0.06	0.14
Prev. line of treatment^b^	74%	1.7 ± 0.8	2.1 ± 1.2	0.36	< .0001	1.8 ± 1.1	1.8 ± 2.0	0.00	0.86
Statin, *n* (%)	100%	572 (58.5%)	267 (60.0%)	0.03	0.61	554 (56.7%)	255 (57.3%)	0.01	0.83
ACEi/ARB, *n* (%)	100%	573 (58.6%)	303 (68.1%)	0.20	0.22	579 (59.3%)	282 (63.4%)	0.09	0.04
CCB, *n* (%)	100%	217 (22.2%)	112 (25.2%)	0.07	0.33	219 (22.4%)	84 (18.9%)	0.09	0.03
Beta-blockers, *n* (%)	100%	279 (28.6%)	116 (26.1%)	0.06	0.80	256 (26.3%)	110 (24.7%)	0.04	0.41
Diuretics, *n* (%)	100%	336 (34.4%)	150 (33.7%)	0.01	0.16	328 (33.5%)	134 (30.0%)	0.08	0.07
APT, *n* (%)	100%	442 (45.2%)	219 (49.2%)	0.08	0.74	442 (45.2%)	221 (49.6%)	0.09	0.03

Variables in the two groups are compared before and after inverse probability of treatment weighting (IPTW). *P* values and standardized mean differences (SMD) are shown and only observed data are presented for the intention-to-treat dataset

*BMI* body mass index, *SBP* systolic blood pressure, *DBP* diastolic blood pressure, *FPG* fasting plasma glucose, *HDL* high-density lipoprotein, *eGFR* estimated glomerular filtration rate, *DME* Diabetic Macular oEdema, *MI* myocardial infarction, *CHD* coronary heart disease, *CKD* chronic kidney disease, *TIA* transient ischemic attack, *LVH* left-ventricular hypertrophy, *ACEi* angiotensin-converting enzyme inhibitors, *ARBs* angiotensin receptor blockers, *CCB* calcium channel blockers, *APT* anti-platelet therapies, *GLM* glucose-lowering medications

^a^The combination of SGLT-2 inhibitors was reimbursed only with concomitant metformin and/or insulin treatment

^b^Number of classes of anti-diabetic drugs used by the patients before the initiation of DPP4i or dapagliflozin

### Overall effectiveness

The median (IQR) time between baseline and follow-up observation was 7.5 (6.2–10.4) months. In the entire cohort, HbA1c declined by 0.7 ± 1.1% (from 8.3% to 7.6%; *p* < 0.0001, *n* = 1422), body weight declined by 1.5 ± 4.6 kg (from 79.1 to 77.6 kg; *p* < 0.0001; *n* = 1222), and systolic blood pressure declined by 2.8 ± 1.19.8 mm Hg (from 141.3 to 138.5 mm Hg; *p* < 0.0001; *n* = 949). IPTW yielded balance of main clinical characteristics (Figure S2), including HbA1c target, with all weighted SMD being < 0.10 and with *p* > 0.01. With IPTW, there was no significant difference in the change in HbA1c (ETD 0.12%, *p* = 0.08), body weight (− 0.88 kg, *p* = 0.053), and systolic blood pressure with dapagliflozin versus DPP-4i. MVA showed comparable results on HbA1c, but significantly greater reductions of body weight and SBP with dapagliflozin than with DPP-4i (Table 2).

**Table 2:** Comparative effectiveness analysis of DPP4i and dapagliflozin on secondary outcomes

	Outcome	DPP4i				Dapagliflozin				ETD (SE)	*p*
		*N*	Baseline	Follow-up	Change	*N*	Baseline	Follow-up	Change		
ITT											
Observed	HbA1c	977	8.0 ± 0.8	7.4 ± 0.9	− 0.6 ± 1.0^	445	8.9 ± 1.2	8.0 ± 1.2	− 0.9 ± 1.2^	0.11 (0.06)	0.07
	Weight	821	75.6 ± 14.2	74.7 ± 13.9	− 1.0 ± 3.3^	401	86.1 ± 15.5	83.5 ± 15.8	− 2.6 ± 6.5^	− 1.20 (0.34)	0.001
	SBP	634	139.6 ± 19.0	137.7 ± 17.6	− 1.9 ± 19.3*	315	144.6 ± 20.2	140.2 ± 18.1	− 4.5 ± 20.8^	− 2.73 (1.37)	0.046
IPTW	HbA1c	977	8.3 ± 1.4	7.5 ± 1.1	− 0.8 ± 1.5	445	8.3 ± 1.9	7.6 ± 1.8	− 0.7 ± 1.8	0.12 (0.07)	0.079
	Weight	821	78.2 ± 18.0	77.2 ± 17.5	− 1.1 ± 4.0	401	79.8 ± 24.9	77.9 ± 28.3	− 2.0 ± 16.9	− 0.88 (0.45)	0.053
	SBP	634	141.3 ± 23.3	139.2 ± 22.1	− 2.1 ± 23.5	315	139.7 ± 32.1	136.0 ± 28.2	− 3.7 ± 33.8	− 1.53 (1.47)	0.30
AT											
Observed	HbA1c	770	8.0 ± 0.8	7.3 ± 0.8	− 0.7 ± 1.0^	320	9.0 ± 1.2	7.9 ± 1.1	− 1.1 ± 1.3^	0.12 (0.07)	0.08
	Weight	649	75.8 ± 14.0	74.9 ± 13.8	− 0.9 ± 3.1^	288	86.0 ± 15.8	83.1 ± 16.4	− 2.9 ± 7.2^	− 1.73 (0.42)	< .0001
	SBP	489	139.6 ± 18.9	137.8 ± 17.7	− 1.9 ± 19.4*	227	143.2 ± 19.7	139.3 ± 18.4	− 4.0 ± 20.4*	− 3.46 (1.63)	0.034
IPTW	HbA1c	770	8.4 ± 1.5	7.4 ± 1.1	− 1.0 ± 1.5	320	8.3 ± 2.0	7.5 ± 1.8	− 0.8 ± 2.0	0.14 (0.08)	0.062
	Weight	649	77.7 ± 16.9	76.7 ± 16.6	− 1.0 ± 3.8	288	79.9 ± 25.3	77.4 ± 29.4	− 2.5 ± 17.8	− 1.49 (0.50)	0.003
	SBP	489	141.3 ± 23.0	139.0 ± 21.7	− 2.3 ± 23.7	227	138.0 ± 34.2	134.8 ± 28.3	− 3.2 ± 35.1	− 0.87 (1.57)	0.581

For each comparison and outcome, we report the number of patients, the values (mean and SD) at baseline and follow-up, the change from baseline, and the estimated treatment difference (ETD) with its standard error (SE), along with the respective *p* values. *p < 0.05 versus baseline; ^*p* < 0.001 versus baseline

*SBP* systolic blood pressure, *FPG* fasting plasma glucose, *ITT* intention to treat, *AT* as treated. The pooled ATD from the ten imputed dataset are presented

### Achievement of individualized HbA1c targets

**Fig. 1:**
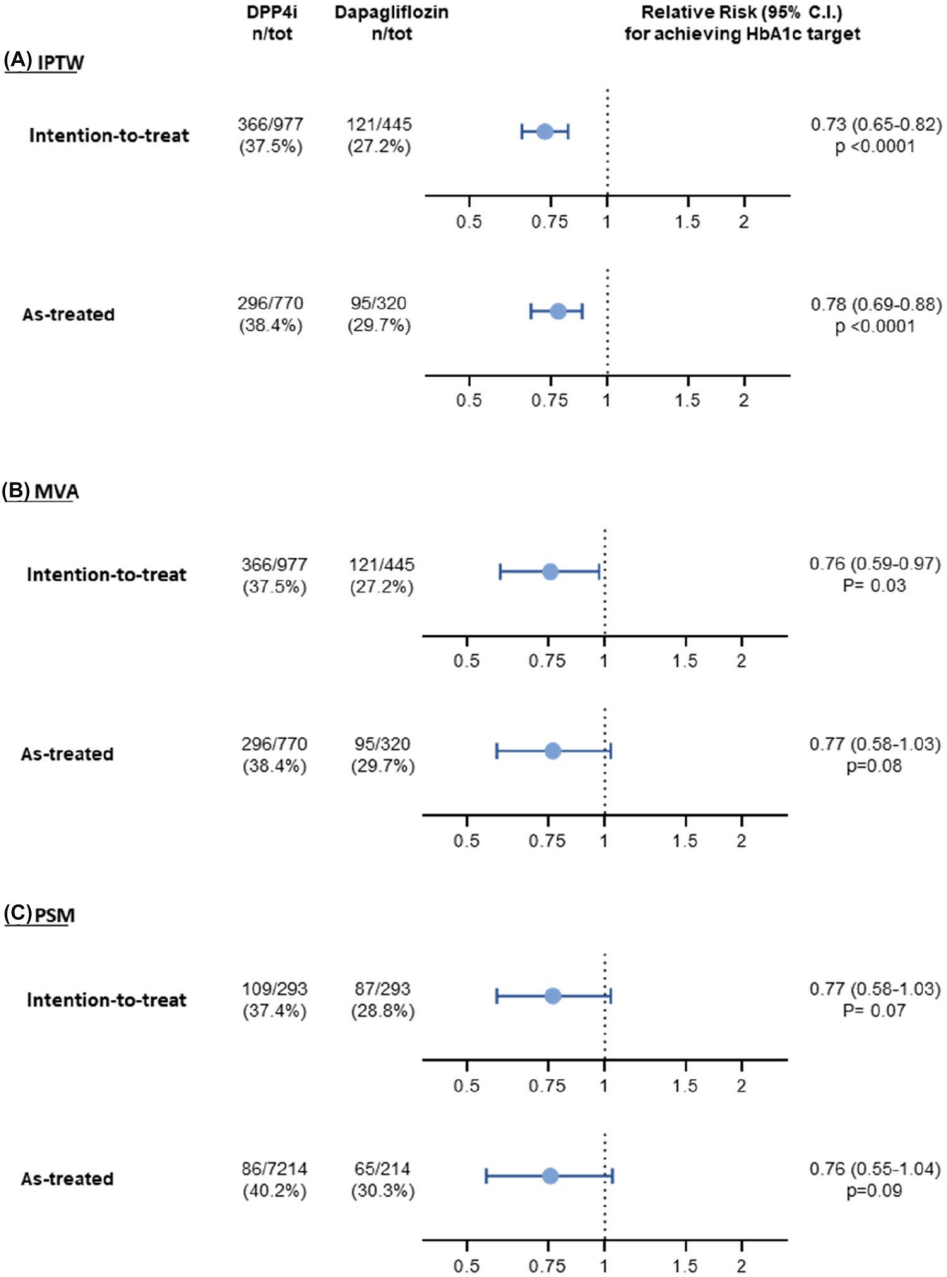
Primary analysis. Probability of reaching the individualized target in initiators of dapagliflozin versus initiators of DPP-4i. **a** Analysis with inverse probability of treatment weighting (IPTW). **b** Analysis with multivariable adjustment (MVA). **c** Analyses with propensity score matching (PSM). The relative risk (RR) is shown with 95% confidence interval (CI) in the intention-to-treat and in the as-treated datasets separately

We calculated individualized HbA1c targets based on the simplified 5-item score [14]. The mean (SD) HbA1c target in this population was 7.1% (0.4%). In the entire cohort, 31.3% of patients achieved such individualized target, and the proportion achieving target was significantly lower with dapagliflozin (27.2%) as compared to DPP4i (37.5%), yielding a rate ratio of 0.73, (*p* < 0.0001), with similar results being observed with IPTW and MVA (Fig. 1). Using PSM, sample size declined and the difference was no longer significant (rate ratio 0.77: 95% CI 0.58–1.03; *p* = 0.077; Table S1). When using standard targets, the result was similar: RR 0.63 (95% CI 0.49–0.81; *p* = 0.0004) for target 6.5%; RR 0.81 (95% CI 0.72–0.92; *p* = 0.0018) for target 7.0%; RR 0.92 (95% CI 0.86–1.00; *p* = 0.042) for target 7.5%.

Prescription of rescue therapies (i.e., add-on glucose-lowering agents other than SGLT2i and DPP-4i after index date) was not different between groups (dapagliflozin 15.7% vs DPP4i 16.5%, IPTW RR 0.96; 95% CI 0.80–1.14; *p* = 0.60).

### Persistency and as-treated analyses

At the last observation, 71.9% and 78.8% of patients were persistent on dapagliflozin and DPP4i, defined as a refilled prescription by the diabetes specialist (*p* for difference = 0.004). Overall results in the AT cohorts confirmed the results observed in the ITT cohort both on HbA1c changes and achievement of targets.

### Subgroup analyses

**Fig. 2:**
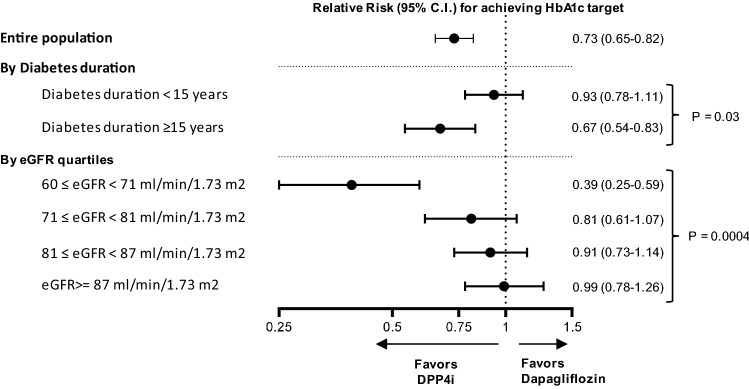
Subgroup analyses. The primary analysis was performed after stratification based on diabetes duration and eGFR quartiles. Results are shown as relative risk (RR) for dapagliflozin versus DPP-4i with 95 confidence interval (CI)

The analysis of effectiveness on achieving the individualized HbA1c target was stratified into several pre-defined variables. The only variables influencing significantly the ETD between dapagliflozin and DPP4i were diabetes duration and eGFR, while all other variables displayed interaction *p* values > 0.1. As shown in Fig. 2, the difference in the proportion of patients achieving HbA1c target was seen only among patients with eGFR close to 60 ml/min/0.173 m^2^ and among those with diabetes duration of 15 years or longer.

## Discussion

Among people with T2D who were aged 70–80 years and who attended Italian diabetes specialist clinics under routine care in 2015–2017, initiation of dapagliflozin was associated with a similar reduction in HbA1c as compared to initiation of DPP-4i, but with a smaller proportion attaining the individualized HbA1c target. This difference was observed mainly among patients with lower eGFR, for whom SGLT2i is known to exert a blunted glycaemic effect [21], due to reduced glucose excretion. In the TriMaster randomized crossover trial, among patients with T2D (mean age 62) and an eGFR between 60 and 90 ml/min/1.73 m^2^, sitagliptin reduced HbA1c more than canagliflozin [22]. In our study, patients with longer diabetes duration also reached HbA1c target less frequently with dapagliflozin than with DPP-4i.

The extra-glycaemic benefits appear to be preserved in older dapagliflozin-treated patients, who experienced greater improvements in blood pressure and reduction in body weight than initiators of DPP-4i. These are important endpoints, as blood pressure control remains an unmet need in elderly individuals with diabetes [23]. Though weight loss may not always be beneficial in the elderly, therapy with SGLT2i is expected to result in loss of adipose and ectopic fat [24, 25], possibly enhancing the overall metabolic improvement. Results on the overall efficacy of dapagliflozin versus placebo are re-assuring on the possibility that SGLT2i maintain their cardiovascular protective effects in elderly patients under routine care, as demonstrated in the stratified analysis of the DECLARE trial [10]. This is supported by results of other trials showing superiority of dapagliflozin versus placebo with regards to heart failure and CKD outcomes including a proportion of elderly patients with and without T2D [7, 9, 26]. For example, in the DELIVER trial, mean age was 72 years and 2806 patients had T2D [26].

It should be noted that the average characteristics of patients included in this study are those of an aged population with advanced and poorly controlled T2D. This scenario differs from that of elderly onset T2D, for whom the development of chronic complications may be less of a concern. Indeed, aged patients with > 10 year diabetes duration, an HbA1c of 8.3%, and highly prevalent complications should be considered particularly at risk of developing adverse diabetes-related outcomes, including heart failure and CKD. Use of SGLT2i in this population is particularly appropriate for the potential to improve disease-related outcomes beyond glycaemic control. Therefore, decisions on the best treatment strategy for this population of patients should not be limited to the evaluation of glycaemic targets. Here, we show that dapagliflozin maintained its effectiveness on extra-glycaemic endpoints in the aged population. Although we do not have data on hard endpoints, it is arguable that simultaneous improvements in glucose, weight, and blood pressure control could translate into improved cardiovascular outcomes. On the other side, no substantial improvement in hard endpoints is expected during treatment with DPP-4i. Therefore, despite a greater proportion of patients attained glycaemic targets with DPP-4i, extra-glycaemic effects were negligible and DPP-4i provide no protection against cardio-renal disease. Therefore, while DPP-4i maintained efficacy in the elderly and are generally well tolerated, we argue that they may be more suited for the treatment of elderly onset diabetes when prevention of heart failure and kidney disease is less of a concern. For elderly patients with high risk for cardio-renal disease and in need of intensifying the glucose-lowering regimen, SGLT2i remain the best option as suggested by treatment algorithms. The combination of SGLT2i and DPP4i is rationale and may be particularly effective and safe [27], also in the elderly.

We did not have information on adverse events, which can be particularly relevant in elderly patients. We observed a persistence of 71.1% on dapagliflozin, which was lower than that observed for DPP-4i (78.8%), but in line with the general population of the DARWIN-FUP study [15], suggesting no specific safety issue leading to treatment discontinuation in this elderly population. Previously, using a similar database, we detected no specific clinical feature leading to discontinuation in patients receiving dapagliflozin, as opposed to those receiving a range of different glucose-lowering medications [28].

We wish to underline that our findings are divergent from those of head-to-head comparative trials. Though with some differences, dapagliflozin and empagliflozin appeared to be non-inferior to saxagliptin and linagliptin, respectively [29, 30], but patients in both trials were much younger than in our study and baseline HbA1c was quite diversified (7.9% and 8.9%) with no use of individualized targets. The reasons why a smaller proportion of patients attained HbA1c targets with dapagliflozin versus DPP-4i in our study probably reside in its specific design and setting. First, there are limitations inherent to the observational design. Though we carefully addressed confounding by indication with gold standard methodologies for comparative effectiveness research, the risk of residual confounding is high, especially because the two populations of patients were very different at baseline. Despite good matching on baseline HbA1c, the higher variability of baseline HbA1c in the dapagliflozin group may be one of the reasons why the proportion of patients reaching HbA1c targets was lower than in the DPP-4i group, even without differences in the change of HbA1c on a continuous scale. Furthermore, systematic factors driving baseline differences between the two groups may have influenced the outcome. During the study period, different reimbursement restrictions, with regards to possible combinations and upper limit of baseline HbA1c, applied to the two classes of drugs, creating a true channelling bias. Therefore, generalizability of our findings needs to be carefully scrutinized.

The glucose-lowering effect of SGLT2i is proportional to eGFR [21] and is supposed to be independent from beta cell function. Thus, it remains unclear why longer diabetes duration was associated with lower proportions of patients attaining HbA1c targets among patients initiated on dapagliflozin versus DPP-4i. While we cannot exclude this finding is due to residual confounding, an inverse association between diabetes duration and glycaemic effectiveness of SGLT2i was noted in prior real-world studies [31, 32], and deserves future investigation. It should also be mentioned that, during the study period, SGLT2i and DPP4i could be prescribed only by diabetes specialists making results not immediately transferrable to primary care. Finally, we acknowledge that the duration of observation was short (7.5 months on average) and a longer follow-up may provide different data with regards to persistence of the glucose-lowering effect of the two treatments.

Nonetheless, our data contribute to building evidence on the effectiveness of SGLT2i in elderly patients with T2D. We detected that a smaller proportion of patients attained individualized targets with dapagliflozin than with DPP-4i, but extra-glycaemic effects of dapagliflozin were preserved in this elderly population and were stronger than those exerted by DPP-4i. In view of the expected benefits of SGLT2i on hard outcomes in this specific population, we advocate for randomized controlled trials dedicated to elderly people with T2D.

## Supplementary Information

Below is the link to the electronic supplementary material.

Supplementary file1 (DOCX 85 KB)

## Data Availability

Restrictions apply to the availability of original data used in this study. Requests can be sent to the corresponding author.
