# Supplementary material for: Short-term effectiveness of dapagliflozin versus DPP-4 inhibitors in elderly patients with type 2 diabetes: a multicentre retrospective study
Source: J Endocrinol Invest. 2023 Jan 9;46(7):1429–39. doi: 10.1007/s40618-022-02002-2 (PMC10261249; doi:10.1007/s40618-022-02002-2)
Supplement: Supplementary file 1 — Supplementary file1 (DOCX 85 KB) [file 40618_2022_2002_MOESM1_ESM.docx]

**ONLINE SUPPLEMENT**

**Composition of the DARWIN-FUP study network.** Federica Ginestra, Gloria Formoso, Agostino Consoli (UOC Endocrinologia e Malattie del Metabolismo Ospedale di Pescara). Francesco Andreozzi, Giorgio Sesti (Medicina Interna, Dipartimento di Scienze Mediche e Chirurgiche Università Magna Graecia di Catanzaro Catanzaro). Salvatore Turco (Servizio di Diabetologia - Centro di Medicina Preventiva, Quarto, Napoli). Luigi Lucibelli (Ambulatorio di Diabetologia, ASL3 Napoli 3 Sud, distretto 56). Adriano Gatti (U.O. Malattie del Metabolismo, Presidio Ospedaliero San Gennaro, Napoli). Raffaella Aldigeri, Alessandra Dei Cas (Endocrinologia Azienda Ospedaliera Universitaria di Parma). Giuseppe Felace (SOC Medicina Interna Spilimbergo, Pordenone). Patrizia Li Volsi (S.S.D. di Endocrinologia e Malattie del metabolismo, ASFO Pordenone). GianPio Sorice, Andrea Giaccari (Endocrinologia e Diabetologia, Policlinico Universitario Agostino Gemelli, Università Cattolica del Sacro Cuore, Roma). Carmen Mignogna, Raffaella Buzzetti (U.O. Dipartimentale di Diabetologia, Dipartimento di Medicina Sperimentale, Azienda Ospedaliero-Universitaria Policlinico Umberto I, Sapienza Università di Roma, Roma). Tiziana Filardi, Susanna Morano (U.O.S. Diabetes Complications, Clinica Medica V, Azienda Ospedaliera-Universitaria Policlinico Umberto I, Dipartimento di Medicina Sperimentale, Università di Roma “La Sapienza”). Ilaria Barchetta, Maria Gisella Cavallo (UOS Diabetologia; Azienda Ospedaliera Policlinico Umberto, Università di Roma “La Sapienza”, Roma). Ilaria Malandrucco, Simona Frontoni (UOC Endocrinologia, Diabetologia e Malattie Metaboliche Ospedale Fatebenefratelli Isola Tiberina Roma). Silvia Carletti, Paola D’Angelo (UOC Diabetologia - Ospedale Sandro Pertini). Gaetano Leto, Frida Leonetti (UOC Diabetologia Universitaria dell'Ospedale SM Goretti, Latina). Paola Silvia Morpurgo, Paolo Fiorina (S.S.D. Malattie Endocrine – Diabetologia, ASST Fatebenefratelli Sacco, P.O. Fatebenefratelli e Oftalmico, Milano. Università degli Studi di Milano). Eva Palmieri, Emanuela Orsi (UO Endocrinologia e Malattie Metaboliche, Fondazione IRCCS Ca' Granda-Ospedale Maggiore Policlinico di Milano). Enzo Mantovani (SSD Diabetologia e Malattie Metaboliche, Presidio Ospedaliero di Mantova). Ivano Franzetti (SSD Endocrinologia-Diabetologia, ASST Valle Olona). Fabrizio Querci (Diabetologia, ASST Bergamo Est). Antonio Bossi (Diabetologia, ASST Bergamo Ovest). Federica Turchi (UOC Diabetologia e Malattie Metaboliche INRCA Ancona). Silvana Manfrini (U.O.S.D Diabetologia e Malattie Metaboliche, Senigallia). Danila Guida, Giuseppe Placentino (SOSD Endocrinologia e Diabetologia, ASL VCO). Guglielmo Beccuti, Fabio Broglio (S.C.D.U. Endocrinologia, Diabetologia e Malattie del Metabolismo A.O.U. Città della Salute e della Scienza di Torino - Università degli Studi di Torino). Franco Cavalot (SSD Malattie Metaboliche e Diabetologia, A.O.U. San Luigi Gonzaga, Orbassano, Torino). Alessandro Nuzzo, Gianluca Aimaretti (Endocrinologia, Azienda Ospedaliera di Novara). Olga Lamacchia (Endocrinology Unit, Department of Medical and Surgical Sciences, University of Foggia). Angelo Cignarelli, Luigi Laviola, Francesco Giorgino (Section of Internal Medicine, Endocrinology, Andrology and Metabolic Diseases; Division of Endocrinology, University Hospital Policlinico Consorziale, Bari). Eleonora Devangelio (Ambulatorio e Day Service di Diabetologia, DSS2 ASL Taranto). Giuliana Cazzetta (ASL Lecce - DSS Gagliano del Capo – Tricase, Lecce). Roberta Chianetta, Roberto Citarrella (Policlinico Universitario di Palermo). Andrea Tumminia, Lucia Frittitta (Centro di Diabetologia, Azienda Ospedaliera Garibaldi, Catania). Massimiliano Anzaldi, Massimo Buscema (U.O.C. di Endocrinologia e Malattie del Ricambio e della Nutrizione, A.O.E. Cannizzaro - Catania). Salvatore Piro, Antonino Di Pino, Francesco Purrello (UOC Medicina Interna, Azienda Ospedaliera Garibaldi, Catania). Antonino Di Benedetto, Giuseppina Russo (Azienda Ospedaliera Universitaria di Messina). Roberto Anichini (U.O. Diabetologia USL Centro, Area Pistoiese). Anna Solini (Department of Surgical, Medical, Molecular and Critical Area Pathology University of Pisa). Monia Garofolo, Stefano Del Prato (UO Malattie Metaboliche e Diabetologia; Azienda Ospedaliera Universitaria Pisana). Bruno Fattor (Servizio di Diabetologia, Ospedale Centrale di Bolzano, Azienda Sanitaria Alto Adige). Gian Paolo Fadini, Angelo Avogaro (UOC Malattie del Metabolismo, Azienda Ospedale Università di Padova). Annunziata Lapolla, Giovanni Sartore (Servizio di Diabetologia ULSS6, Padova). Michele D’Ambrosio, Virgilio Da Tos (UOSD Diabetologia, ULSS6 Monselice, Padova). Vera Frison, Natalino Simioni (Medicina Interna e Diabetologia, Ospedale di Cittadella, Padova). Massimo Cigolini, Enzo Bonora (UOC Endocrinologia, Diabetologia e Malattie Del Metabolismo, Azienda Ospedaliera Universitaria Integrata di Verona). Elisabetta Brun, Marco Strazzabosco, (UOC Malattie Endocrine, del Ricambio e della Nutrizione, AULSS 8 Berica). Maurizio Poli (Servizio di Diabetologia, Ospedale San Bonifacio, AULSS9 Verona). Mauro Rigato, Agostino Paccagnella (UOC Malattie Endocrine, del Ricambio e della Nutrizione Azienda AULSS 2 della Marca Trevigiana). Carmela Vinci (Servizio di Diabetologia, ULSS4 Veneto).

**Table S1. Clinical characteristics of study patients ITT dataset**. Variables in the two groups are compared after propensity score matching. P-values and standardized mean differences (SMD) are shown and only observed data are presented for the intention-to-treat dataset. Data shown are the average across the 10 imputed datasets.

|  | **After PSM intention-to-treat** | | | | **After PSM as-treated** | | | |
| --- | --- | --- | --- | --- | --- | --- | --- | --- |
|  | **DPP4i**  **N=293** | **Dapagliflozin N=293** | **SMD** | **p** | **DPP4i**  **N=214** | **Dapagliflozin N=214** | **SMD** | **p** |
| Year Visit | 2017 (2016-2017) | 2016 (2016-2017) | 0.02 | 0.85 | 2017 (2016-2017) | 2016 (2016-2017) | 0.01 | 0.99 |
| Age, years | 73.5 ± 2.9 | 73.4 ± 2.8 | 0.03 | 0.71 | 73.3 ± 2.8 | 73.4 ± 2.7 | 0.03 | 0.78 |
| Sex male, n (%) | 172 (58.6%) | 168 (57.2%) | 0.03 | 0.76 | 134 (62.6%) | 129 (60.4%) | 0.05 | 0.67 |
| Diabetes duration, years | 13 (8 - 20) | 14 (8 - 20) | 0.06 | 0.52 | 13 (8 - 19) | 13 (8 - 21) | 0.04 | 0.73 |
| Weight, kg | 82.5 ± 15.1 | 82.1 ± 13.7 | 0.01 | 0.89 | 82.0 ± 15.1 | 82.1 ± 13.7 | 0.01 | 0.93 |
| BMI, kg/m^2^ | 30.1 ± 5.0 | 30.1 ± 4.5 | 0.02 | 0.78 | 29.9 ± 5.2 | 29.9 ± 4.5 | 0.01 | 0.97 |
| SBP, mm Hg | 141.8 ± 20.0 | 142.7 ± 19.5 | 0.04 | 0.66 | 140.9 ± 19.6 | 141.9 ± 19.7 | 0.05 | 0.63 |
| DBP, mm Hg | 77.1 ± 9.1 | 77.1 ± 9.7 | 0.02 | 0.85 | 76.6 ± 9.1 | 76.8 ± 9.5 | 0.02 | 0.78 |
| FPG, mg/dl | 167.6 ± 47.6 | 170.0 ± 50.2 | 0.03 | 0.81 | 168.0 ± 48.8 | 170.1 ± 50.7 | 0.04 | 0.65 |
| HbA1c, % | 8.5 ± 1.1 | 8.6 ± 1.0 | 0.08 | 0.36 | 8.5 ± 1.1 | 8.6 ± 1.0 | 0.12 | 0.25 |
| Target HbA1c, (%) | 7.2 ± 0.4 | 7.2 ± 0.4 | 0.04 | 0.68 | 7.2 ± 0.4 | 7.2 ± 0.4 | 0.10 | 0.36 |
| At target, n (%) | 0 (0.0%) | 0 (0.0%) |  |  | 0 (0.0%) | 0 (0.0%) |  |  |
| Total cholesterol, mg/dl | 168.2 ± 38.8 | 168.9 ± 33.5 | 0.01 | 0.96 | 168.9 ± 39.9 | 169.4 ± 33.3 | -0.02 | 0.98 |
| HDL cholesterol, mg/dl | 49.9 ± 13.4 | 49.3 ± 13.5 | 0.02 | 0.80 | 49.8 ± 13.7 | 49.8 ± 14.1 | 0.00 | 0.91 |
| Triglycerides, mg/dl | 120 (90 - 171) | 125 (90 - 169) | 0.03 | 0.78 | 124 (87 - 175) | 123 (89 - 168) | -0.02 | 0.79 |
| LDL cholesterol, mg/dl | 91.6 ± 33.3 | 91.5 ± 28.2 | 0.00 | 0.98 | 92.5 ± 33.7 | 92.1 ± 28.4 | 0.01 | 0.91 |
| eGFR, ml/min/1.73 m^2^ | 80.4 ± 10.3 | 80.3 ± 10.2 | 0.01 | 0.92 | 81.0 ± 10.3 | 80.7 ± 10.3 | 0.03 | 0.75 |
| Complications |  |  |  |  |  |  |  |  |
| CKD III stage, n (%) | 0 (0.0%) | 0 (0.0%) |  |  | 0 (0.0%) | 0 (0.0%) |  |  |
| Nephropathy, n (%) | 49 (16.7%) | 51 (17.4%) | 0.02 | 0.83 | 34 (15.9%) | 36 (16.9%) | -0.03 | 0.78 |
| Retinopathy, n (%) | 39 (16.7%) | 39 (16.2%) | 0.00 | 0.91 | 27 (15.8%) | 28 (16.4%) | -0.02 | 0.94 |
| DME, n (%) | 6 (2.4%) | 7 (2.7%) | 0.00 | 0.81 | 4 (2.3%) | 5 (2.9%) | -0.04 | 0.82 |
| Carotid ather., n (%) | 68 (40.4%) | 63 (38.6%) | 0.03 | 0.74 | 52 (42.4%) | 46 (39.1%) | 0.07 | 0.85 |
| Stroke/TIA, n (%) | 12 (6.9%) | 13 (8.1%) | 0.02 | 0.84 | 10 (7.8%) | 10 (8.9%) | -0.04 | 0.61 |
| Prior MI, n (%) | 12 (5.0%) | 13 (4.9%) | 0.00 | 0.97 | 10 (5.5%) | 10 (5.6%) | 0.00 | 0.90 |
| Coronary Revasc., n (%) | 17 (6.9%) | 19 (7.3%) | 0.02 | 0.80 | 14 (7.9%) | 15 (8.0%) | 0.00 | 1.00 |
| CHD, n (%) | 26 (10.5%) | 29 (11.4%) | 0.03 | 0.72 | 21 (11.6%) | 22 (12.0%) | -0.01 | 0.95 |
| Heart Failure. n (%) | 5 (2.0%) | 7 (2.6%) | 0.03 | 0.71 | 3 (1.5%) | 4 (1.9%) | -0.04 | 0.82 |
| LVH, n (%) | 21 (8.3%) | 26 (10.0%) | 0.03 | 0.73 | 14 (8.0%) | 17 (9.4%) | -0.05 | 0.84 |
| Microangiopathy, n (%) | 108 (36.8%) | 111 (37.9%) | 0.08 | 0.81 | 73 (34.2%) | 76 (35.4%) | -0.03 | 0.80 |
| Macroangiopathy, n (%) | 98 (38.1%) | 95 (36.8%) | 0.02 | 0.89 | 72 (38.5%) | 72 (38.7%) | 0.00 | 0.97 |
| Established CVD, n (%) | 43 (16.9%) | 45 (17.3%) | 0.01 | 0.78 | 35 (18.5%) | 36 (19.5%) | -0.02 | 0.76 |
| Diabetes medications* |  |  |  |  |  |  |  |  |
| Insulin, n (%) | 144 (49.1%) | 149 (50.8%) | 0.01 | 0.73 | 99 (46.1%) | 111 (52.0%) | -0.12 | 0.28 |
| Metformin, n (%) | 266 (90.9%) | 265 (90.6%) | 0.01 | 0.92 | 195 (90.9%) | 194 (90.6%) | 0.01 | 0.93 |
| Prev. line of treatment§ | 1.9 ± 0.9 | 1.9 ± 1.1 | 0.01 | 0.87 | 1.9 ± 0.9 | 1.9 ± 1.1 | -0.06 | 0.75 |
| Statin, n (%) | 165 (56.4%) | 167 (57.0%) | 0.00 | 0.90 | 130 (60.6%) | 130 (60.6%) | 0.00 | 1.00 |
| ACEi/ARB, n (%) | 183 (62.6%) | 184 (62.9%) | 0.00 | 0.94 | 131 (61.4%) | 132 (61.5%) | 0.00 | 0.98 |
| CCB, n (%) | 65 (22.3%) | 66 (22.6%) | 0.01 | 0.94 | 49 (22.7%) | 52 (24.4%) | -0.04 | 0.70 |
| Beta-blockers, n (%) | 75 (25.6%) | 74 (25.5%) | 0.00 | 0.97 | 56 (26.1%) | 59 (27.5%) | -0.03 | 0.76 |
| Diuretics, n (%) | 92 (31.4%) | 93 (31.7%) | 0.01 | 0.94 | 68 (31.9%) | 66 (30.7%) | 0.03 | 0.79 |
| APT, n (%) | 134 (45.8%) | 135 (46.2%) | 0.01 | 0.93 | 99 (46.3%) | 97 (45.1%) | 0.02 | 0.82 |

BMI, body mass index. SBP, systolic blood pressure. DBP, diastolic blood pressure. FPG, fasting plasma glucose. HDL, high density lipoprotein. eGFR, estimated glomerular filtration rate. DME, Diabetic Macular Edema. MI, Myocardial Infarction. CHD, Coronary heart disease. CKD, chronic kidney disease. TIA, transient ischemic attack. LVH, left ventricular hypertrophy. ACEi, angiotensin converting enzyme inhibitors. ARBs, angiotensin receptor blockers. CCB, calcium channel blockers. APT, anti-platelet therapies. GLM, glucose lowering medications. *The combination of SGLT-2 inhibitors were reimbursed only with concomitant metformin and/or insulin treatment. §Number of classes of anti-diabetic drugs used by the patients before the initiation of DPP4i or Dapagliflozin.

**Figure S1.** Study flowchart (*Average of patients included in PSM dataset across 10 imputed dataset).


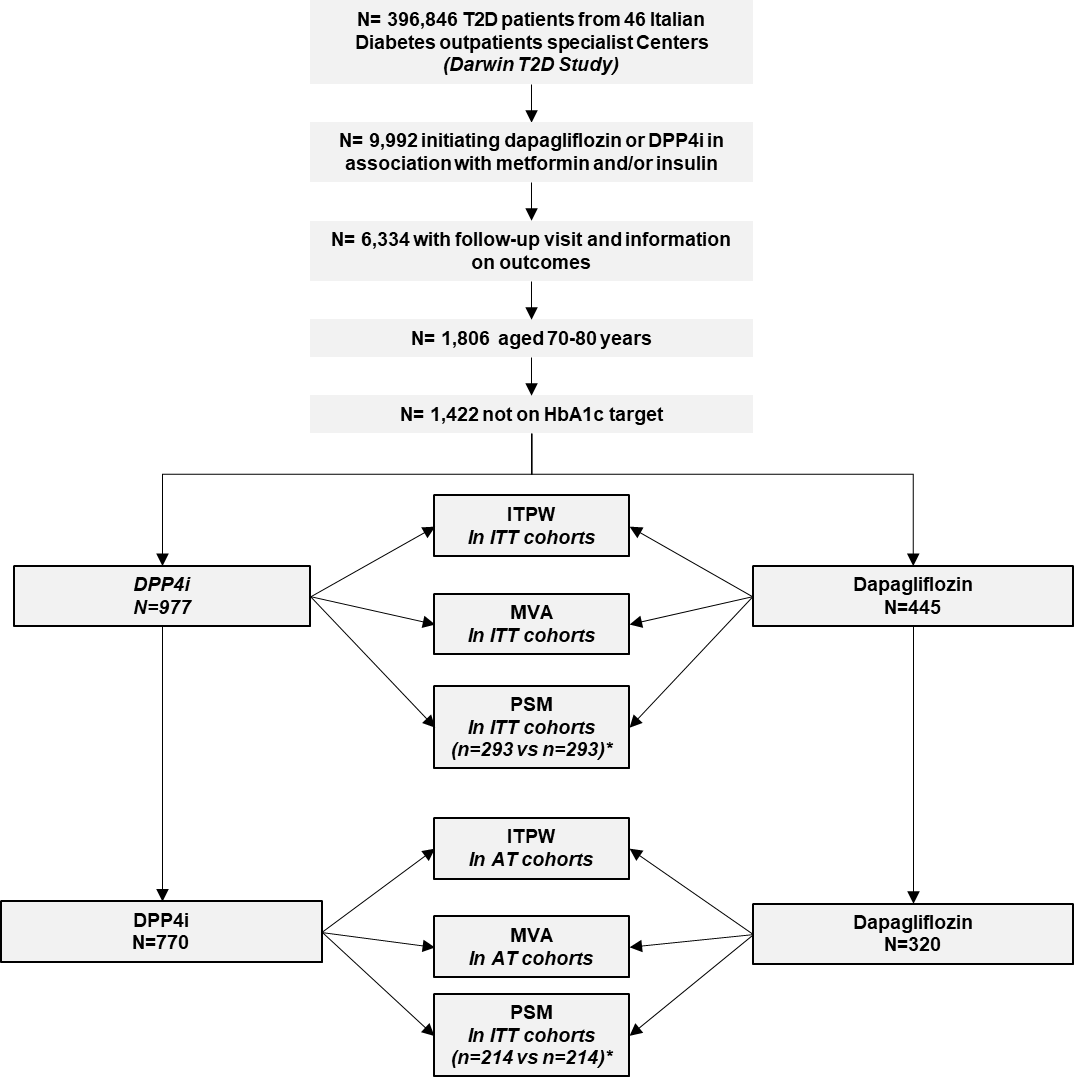


**Figure S2**. Covariate balance before and after inverse probability of treatment weighting (IPTW). Analysis performed in the intention-to-treat dataset.
